# Virtual reality as a non-pharmacologic analgesic for fasciotomy wound infections in acute compartment syndrome: a case report

**DOI:** 10.1186/s13256-020-02370-4

**Published:** 2020-04-14

**Authors:** Ryo Esumi, Ayumu Yokochi, Motomu Shimaoka, Eiji Kawamoto

**Affiliations:** 1 Department of Emergency and Disaster Medicine, Mie University Graduate School of Medicine, 2-174 Edobashi, Tsu, Mie 514-8507 Japan; 2 Department of Anesthesiology and Critical Care Medicine, Mie University Graduate School of Medicine, 2-174 Edobashi, Tsu, Mie 514-8507 Japan; 3 Department of Molecular Pathobiology and Cell Adhesion Biology, Mie University Graduate School of Medicine, 2-174 Edobashi, Tsu, Mie 514-8507 Japan

**Keywords:** Virtual reality, Fasciotomy wound infections, Acute compartment syndrome

## Abstract

**Background:**

Fasciotomy is a life-saving procedure to treat acute compartment syndrome, a surgical emergency. As fasciotomy dramatically improves wound pain, it should be performed as soon as possible. Moreover, delays in the use of fasciotomy can increase the rate of wound infections. Once the fasciotomy wound is infected, pain control is achieved via the long-term use of opioids or anti-inflammatory analgesics. However, the administration of high doses of opioids may cause complications, such as respiratory depression, over-sedation, and constipation. Therefore, treatment methods other than narcotic administration should be established to better manage the pain caused by fasciotomy wound infections.

Virtual reality has recently been introduced in analgesic therapy as a replacement, or complement, to conventional pharmacological treatments. Its use has been extensively studied in the pain management of patients with burns. An increasing number of painful conditions are being successfully treated with virtual reality. Here, we report a case of acute compartment syndrome complicated by fasciotomy wound infection.

**Case presentation:**

A 40-year-old Japanese man suffering from acute compartment syndrome of his leg due to a car accident trauma was treated with a fasciotomy to decompress intra-compartmental pressure and restore tissue perfusion, and admitted to an intensive care unit. Unfortunately, as the open fasciotomy wound was complicated by infection, he complained of hyperalgesia and severe pain during wound debridement. He was therefore given acetaminophen and high-dose intravenous patient-controlled analgesic fentanyl (35 μg/kg per day) to reduce the pain. Despite these efforts, the pain was poorly controlled and opioid-induced side effects such as respiratory depression were observed. An immersive virtual reality analgesic therapy aimed at distraction and relaxation was used and effectively alleviated the pain. Three sessions of virtual reality analgesic therapy over 2 days produced sustainable analgesic effects, which led to a 25–75% dose reduction in fentanyl administration and the concomitant alleviation of respiratory depression.

**Conclusions:**

This case suggests the feasibility of virtual reality analgesic therapy for pain management of fasciotomy wound complications in acute compartment syndromes. Virtual reality represents a treatment option that would reduce analgesic consumption and eliminate opioid-induced respiratory depression to treat fasciotomy wound infection.

## Introduction

Acute compartment syndrome (ACS) is a serious complication of limb trauma, in which the swelling of injured tissues and/or concomitant hematoma causes an increase in intra-compartmental pressure, thereby constraining blood perfusion of the tissues [1]. Ischemia induces neurological symptoms such as numbness and pain. If low or lack of blood perfusion persists, it can ultimately cause tissue necrosis, irreversibly damaging the limbs [1, 2].

A prompt execution of fasciotomy is recommended to decompress the intra-compartmental pressure and restore tissue perfusion, once a severe rise in pressure is either confirmed by invasive monitoring or assessed from clinical symptoms [3]. Although early fasciotomy is recommended for those suffering from ACS, this invasive procedure leaves the wound open, thereby endangering patients with potential complications such as wound infections, which have been reported to occur in 10–30% of cases [4, 5].

Severe pain is a hallmark of ACS and strongly suggests the presence of acutely increased intra-compartmental pressure and resulting tissue ischemia, thereby prompting the need for a decompressive fasciotomy [1, 2]. When decompression of the intra-compartmental pressure is achieved by fasciotomy, the intensity of the pain should gradually cease. A short course of opioid treatment is then suitable for pain management in patients with ACS [6]. However, pain can be recurrent and can even worsen when a fasciotomy wound is compounded by infection, thereby complicating the use of opioids for pain management [7–9].

Virtual reality (VR) has recently emerged as a novel analgesic therapy that could replace or complement conventional pharmacological treatments, and has been extensively studied in the pain management of patients with burns. The list of painful conditions successfully treated with VR is growing. Here we add to the list a case of ACS complicated by fasciotomy wound infection. In this case, the patient’s pain was difficult to manage with opioids due to intolerable adverse effects such as nausea and respiratory depression.

## Case presentation

A 40-year-old Japanese man, a truck driver, suffered multiple traumas during a road car crash that severely damaged the front part of his truck. While he was trapped in the driver’s seat, his lower-right limb was strongly pinched against the dashboard for 8 hours until he was saved by a rescue team. He was then transferred to an intensive care unit (ICU) at Mie University Hospital, a tertiary academic medical care center.

**Fig. 1:**
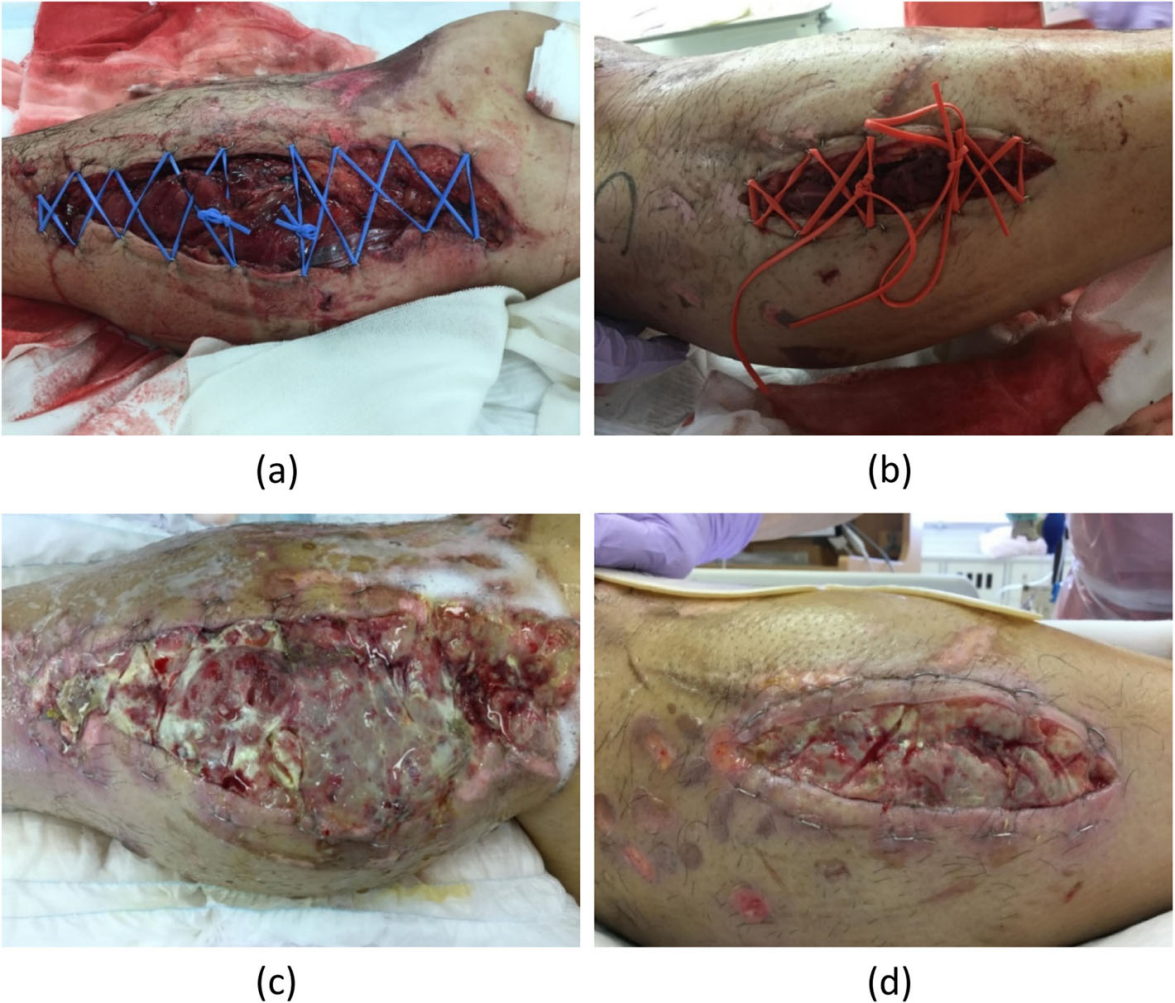
Clinical images of right foot acute compartment syndrome in a 40-year-old man injured in a road car accident. (*Top*) Images taken immediately after the fasciotomy on day 1; (*bottom*) images taken on day 8, showing wound infections. (**a**) and (**c**) indicate the inside of the lower leg. (**b**) and (**d**) indicate the outside of the lower leg

He was fully alert and complained of severe pain, along with numbness and weakness, in his right limb. A full-body computed tomography scan revealed multiple rib and lumbar compression fractures. His right lower leg had no fractures; however, the muscles in his lower leg were significantly swollen after the prolonged compression. After placing intramuscular catheters to monitor the intra-compartment pressures of his lower limb, the trauma team found that the pressures in the anterior, posterior, medial, and lateral side compartments had risen to ~ 50 mmHg. Based on these findings, he was diagnosed as having ACS, and quickly underwent a fasciotomy of his right lower limb. The fasciotomy wounds were left open (Fig. 1 top panels), and were cleaned daily and wrapped in a dressing containing an antibiotic ointment.

**Fig. 2:**
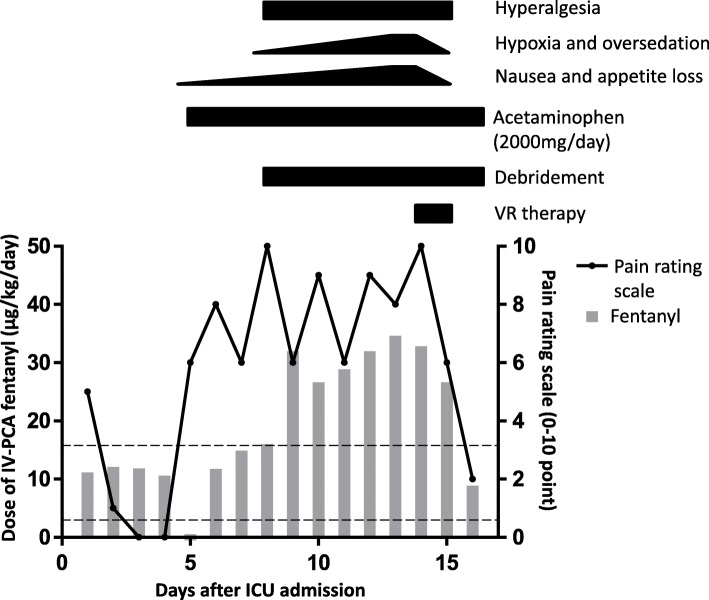
Clinical course of intravenous patient-controlled analgesia fentanyl using a 0–10 pain rating scale. On days 14 and 15, the patient was administered an immersive virtual reality. Debridement of infected necrotic tissues was performed beginning on day 8. The dotted line represents the appropriate continuous intravenous infusion of fentanyl (2.88–16.08 μg/kg per day). The time courses for the major clinical events and approximate intensities of the opioid-induced adverse effects are shown. *ICU* intensive care unit, *IV-PCA* intravenous patient-controlled analgesia, *VR* virtual reality

After the fasciotomy, the neurological deficits of his right limb were gradually restored and the pain intensity was reduced, made manageable by opioid treatment for at least for 4 days (Fig. 2).

**Fig. 3:**
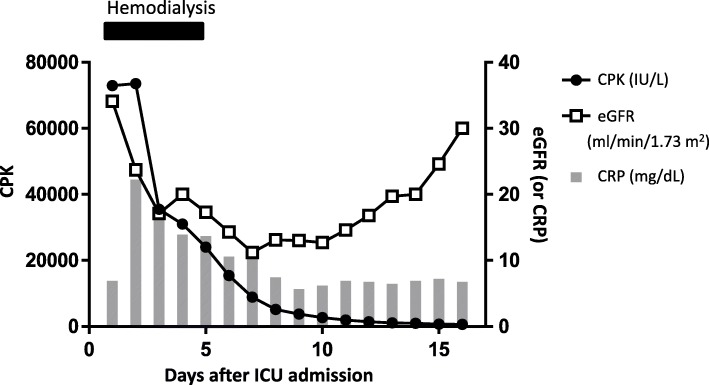
Time course of rhabdomyolysis (creatine phosphokinase), acute renal failure (estimated glomerular filtration rate), and inflammation (C-reactive protein). Hemodialysis was performed only for the first 5 days. Creatine phosphokinase values are indicated on the left axis, the estimated glomerular filtration rate values are indicated on the right axis, and C-reactive protein values are indicated on the right axis. *CPK* creatine phosphokinase, *CRP* C-reactive protein, *eGFR* estimated glomerular filtration rate, *ICU* intensive care unit

He exhibited rhabdomyolysis with increased levels of serum creatine phosphokinase (CPK) and acute renal failure, possibly due to the ischemia reperfusion injury associated with ACS (Fig. 3). His CPK level progressively decreased and returned to normal levels on day 10, indicating resolution of the rhabdomyolysis. Acute renal failure temporarily required hemodialysis for 5 days, and he subsequently recovered.

The pain at the fasciotomy wounds on his right leg was well managed for 4 days (days 1 to 4), as shown by the score of 5 points on the Numeric Rating Scale (NRS) for pain (0–10) [10], via intravenous patient-controlled analgesia (IV-PCA) fentanyl at a dose of ~ 12 μg/kg per day (Fig. 2). On day 5, owing to the onset of nausea, IV-PCA fentanyl was discontinued and replaced with a drip infusion of acetaminophen. However, as acetaminophen could not sufficiently control the pain, IV-PCA fentanyl was resumed along with antiemetic agents on day 6. On day 8, as our patient’s pain at the wounds intensified, we observed the appearance of defective granulation and necrotic tissue at the site of the fasciotomy despite adequate infection prevention. In addition, the wounds exuded a foul odor and were strongly suggestive of infection (Fig. 1 bottom panels). To control the wound infections, debridement of the infected necrotic tissues was performed.

Although the wound infections appeared to be under control with antibiotics and debridement, our patient continued to complain of severe pain both during and between procedures, as shown by the NRS of 6–10 points. The pain experienced was self-described as if being stabbed with a pin or a needle or, alternately, a numbness resulting in a dull sensation. The pain began abruptly, irrespective of body movement, and lasted for approximately 1 hour. A dose of IV-PCA fentanyl to manage the pain was progressively increased up to 35 μg/kg per day from day 10 to 13. He also presented hyperalgesia of his right limb beginning at day 10. In addition, nausea and poor appetite worsened as a side effect of high-dose fentanyl, and he experienced loud snoring and excessive daytime sleepiness due to opioid-induced respiratory depression; as such, he was administered oxygen to prevent hypoxia. We consulted with an in-hospital pain control team about a pain management strategy to replace/complement opioid administration. They confirmed the presence of opioid-refractory severe pain and hyperalgesic states, and proposed the use of VR analgesia.

On day 14, he was provided with an immersive VR using the Samsung Gear Oculus headset fitted with a Samsung Galaxy S7 phone loaded with the AppliedVR (AVR) healthcare platform (AppliedVR Inc., Los Angeles, CA 90067, USA), which delivers various VR analgesia program modules. Of 20+ VR programs designed to distract and/or relax, the program “Dream Beach” was selected according to our patient’s preference for the sea. The VR program simulates the experience of being at the beach beside a calm sea on a sunny day. Each session lasted for 30 minutes and three sessions were administered over 2 days. The VR analgesic proved effective, as his pain rating fell dramatically from 10 to 6 points. On day 15, the second day of VR administration, its analgesic effects proved so successful that bolus infusions of IV-PCA fentanyl were no longer required. On day 16, the pain rating scale remained 2 points under a baseline IV-PCA infusion of 8.8 μg/kg per day fentanyl, and as the wound pain became manageable, he was transferred from the ICU to a general surgery ward at a secondary care hospital. At day 28, after the fasciotomy, he was free of opioids and the wounds were closed using split-thickness skin grafting.

## Discussion and conclusions

In this case report, we present a successful application of an immersive VR experience to alleviate severe pain from the open fasciotomy wounds of a patient with ACS who had been treated with a high-dose (that is, 35 μg/kg per day fentanyl) IV-PCA opioid, which became intolerable due to adverse effects such as nausea and respiratory depression. The analgesic effects brought about by this immersive VR experience to our patient made it possible to reduce the doses of IV-PCA fentanyl by 25~75%, which alleviated the opioid-induced respiratory depression. This case study not only confirms previous reports showing the effectiveness of VR to alleviate pain during wound care and physical therapy in patients with burns [11, 12], but also illustrates the feasibility of using a VR analgesic to manage pain in a patient with ACS.

In patients with ACS, the pain during the early phase – that is, within several days after fasciotomy – may stem from physical tissue damage and inflammation, as well as from ischemia and ischemia-reperfusion that can damage neuronal and non-neuronal cells [1, 6]. Generally speaking, pain in the early phase gradually decreases, as inflammation resolves while the wounds heals. Such pain is usually manageable in the early phase with opioids, as occurred in our case [6]. In contrast, pain in the late phase – that is, several days after the fasciotomy – may indicate the presence of complications associated with ACS and fasciotomy, such as wound infections and neuropathic pain [2, 4]. Pain in the late phase is often not well managed with opioids, as was observed in this case. This is partly due to the fact that prolonged use of opioids is accompanied by several adverse effects, from nausea, itching, and constipation to respiratory depression and the development of tolerance and dependency [13]. Of note, opioids have been shown to exhibit a wide range of immune-suppressive effects [14], thereby potentially worsening wound infections [15]. Hyperalgesia also occurred in our case. Hyperalgesia can be divided into three types: primary, secondary, and opioid-induced. Primary hyperalgesia is caused by the exacerbation of pain due to tissue damage. Secondary hyperalgesia involves the spreading to undamaged tissue. In general, pain spreads to the areas surrounding the damaged tissue. Opioid-induced hyperalgesia is thought to be induced by the administration of opioids such as morphine and fentanyl to relieve pain [16]. However, the narcotic side effects can be severe enough to warrant discontinuation of opioid treatment. In the current case, as primary or opioid-induced hyperalgesia may have occurred, it was necessary to reduce the dose of fentanyl as quickly as possible. Thus, it is of great clinical significance that a VR analgesic proved effective in alleviating the pain in the late phase of a patient with ACS treated with fasciotomy, thereby reducing the need for opioids [12].

Continuous infusion of fentanyl at 2.88–16.08 μg/kg per day for several days has often been safely used without any serious side effects such as respiratory depression [17, 18]. The appearance of serious adverse effects such as respiratory depression, which can require intubation, would hamper the continuous use of high-dose fentanyl [19], as was the case in this report. Thus, a pain management approach that would replace/complement opioids, thereby mitigating the risk of opioid-induced respiratory depression, would be extremely useful in ICUs and other clinical settings. In fact, opioid-induced respiratory depression represents the major morbidity associated with opioid abuse [20]. Considering the adverse effects of opioids and the serious negative social impact of widespread opioid addiction that originates from the misuse of prescription drugs [21], alternative means of pain control that can reduce opioid usage are of great clinical importance. VR is a promising non-pharmacological means to replace and/or complement opioids [12]. It has been shown in healthy individuals that the thermal stimulation of VR confers additive analgesic effects to opioid treatment in alleviating pain [22]. Consistent with these findings, our own case of ACS showed that pain relieved by VR resulted in reduced opioid requirements, which alleviated our patient’s opioid-induced respiratory depression. Further investigations would be needed to test how well VR can replace/complement opioid analgesics in acute and chronic pain conditions in various diseases.

The major mechanism by which the VR program described in our case reduced pain was distraction, which is designed to dilute a patient’s attention to pain, supplanted by an immersive VR environment, which modulates a patient’s pain perception [12]. Relaxation, another mechanism closely related to distraction, was also employed in our VR analgesic regimen. As pain perception can be influenced by a patient’s affect (a psychological term describing the experience of positive emotion), shifting the distressing circumstances of being in a wounded state in a hospital room toward the much more enjoyable circumstances of a pleasant VR environment gives rise to a positive effect, which alleviates pain. To optimize the effects of distraction and relaxation, proper selection of the VR content is critically important. In fact, although we prescreened 20+ available VR programs based on our patient’s preference for the sea, the “Dream Beach” program proved effective, while the “Sea Hospital” program, which simulates the experience of being at a pool with seals, was not effective.

Although not applicable to the present case, focus-shifting and skill-building represent two additional advanced mechanisms of VR analgesia [12]. As is often observed in gaming-type VR programs such as “Bear Blast”, which involves a shooting game to target bears with cannon balls, focus-shifting potently shifts one’s attention to VR objects, and requires the user’s focused interaction with a VR environment. It has been shown in patients with burns that VR analgesics based on focus-shifting mechanisms are more effective at alleviating pain than that of mere passive distraction [23]. Skill-building aims to foster a patient’s capacity to achieve a certain mental state, such as mindfulness mediation, in order to control their mental and physical responses to painful conditions [24, 25]. As skill-building would require more active engagement than simple passive distraction [12], this might prove difficult for some patients in ICU. Skill-building VR is expected to be effective for the management of chronic pain [12].

How long the analgesic effects of VR can last represents an important and unresolved question. It has been shown that the preoperative administration of immersive VR experiences made pediatric patients more resilient to postoperative pain [26]. It is possible that VR analgesia, under certain settings, may give rise to sustainable modulation of pain perception. In our case, our patient felt significantly less pain not only during, but also sometime after administration of the VR analgesic, thus suggesting that the effects could last for hours or days. A possible explanation for the long-lasting analgesic effects of VR in our case might be that a VR-induced positive shift of our patient’s affect helped modulate pain perception in a lasting manner, continuing even after the VR session had ended [12].

A potential limitation of VR analgesics is the cost of introducing such a platform (including software and hardware) to clinics. Although the initial expenses might be high, one recent economic analysis using computer modeling has estimated that, overall, VR analgesic therapy would be cost saving whenever it reduced the length of hospitalization [27]. Real-world economic analyses are needed to carefully assess the economic benefits of VR analgesic therapies. In addition, potential adverse effects, if any, might limit the utility of VR analgesia in clinics. Several clinical studies have occasionally, though quite infrequently, reported minor incidences such as nausea, thereby supporting the overall safety profile of VR’s clinical applications [11]. As is sometime seen in recreational VR users, motion sickness, which can induce nausea, is a major factor potentially limiting the utility of VR analgesia [28]. Investigations into human and machine factors affecting susceptibility to nausea, as well as the development of novel technologies mitigating such side effects are currently underway.

## Data Availability

Not applicable.

## Abbreviations

VR: Virtual reality
IV-PCA: Intravenous patient-controlled analgesia
NRS: Numeric Rating Scale
ACS: Acute compartment syndrome
ICU: Intensive care unit
CPK: Creatine phosphokinase
